# Histological and chemical view on parotid duct sialolithiasis in the Slovakian warmblood mare

**DOI:** 10.1007/s11259-024-10485-y

**Published:** 2024-07-31

**Authors:** Filip Korim, Viera Revajová, Filip Koľvek, Lukáš Bujňák, Sebastián Hreus, Dalibor Všianský

**Affiliations:** 1 Department of Morphological Disciplines, University of Veterinary Medicine and Pharmacy in Košice, Komenského 73, Košice, 041 81 Slovak Republic; 2 Equine Clinic, University Veterinary Hospital, University of Veterinary Medicine and Pharmacy in Košice, Komenského 73, Košice, 041 81 Slovak Republic; 3 Department of Animal Nutrition and Husbandry, University of Veterinary Medicine and Pharmacy in Košice, Komenského 73, Košice, 041 81 Slovak Republic; 4 Institute of Geosciences, Faculty of Mining, Ecology, Process Control and Geotechnologies, Technical University of Košice, Park Komenského 15, Košice, 040 01 Slovak Republic; 5 Department of Geological Sciences, Faculty of Science, Masaryk University, Kotlářská 267/2, Brno, 611 37 Czech Republic

**Keywords:** Equine, Histology, Chemistry, Parotid duct, Sialolith

## Abstract

The parotid duct has been reported to be the most common site of sialoliths in horses. In this case report, we described the first confirmed case of the equine sialolithiasis in Slovakia. The work was aimed to describe the transcutaneous approach to removing the sialolith, which manifested as a hard painless mass in the area of the maxillary cheek teeth, in a 14-year-old Slovakian warmblood mare. Pathological-anatomical and histological examination after extirpation confirmed the presence of parotid duct ectasia resulting from calculus. The mineral composition of the sialolith was determined with atomic absorption spectroscopy using X-ray powder diffraction. The sialolith was successfully extirpated transcutaneously, without complications or recurrence.

## Introduction

The parotid salivary gland (*glandula parotis*) is the largest serous salivary gland in the horse. The parotid duct (*ductus parotideus*), which goes through mandibular notch (*incisura vasorum facialium*) with the facial artery and facial vein, transports saliva from the parotid salivary gland. The terminal part of the parotid duct runs on the lateral side of the cheek, opening at the *papilla parotidea* lying in the *vestibulum buccale*, opposite the third premolar (Singh 2017; Orsini et al. 2022).

Sialolithiasis of the parotid duct is an uncommon disease. It presents as a non-painful, hard, mineralized mass, lying rostrally from the facial crest in the level of the third and fourth premolar. Sialolith formation is initiated by the presence of a foreign body (grain) in the parotid duct. The foreign body initiates an inflammatory reaction with or without the microbiota, resulting in calcium salts (calcium carbonate, calcium oxalate and calcium phosphate) accumulating around it (Kay 2006; Al-Sobayil and Ibrahim 2008; Oreff et al. 2016; Poore et al. 2019; Sadaksharam and Kuduva Ramesh 2020). However, the precise aetiology of sialolithiasis is still unknown (Al-Sobayil and Ibrahim 2008; Sadaksharam and Kuduva Ramesh 2020).

This study aims to describe the first confirmed case of the sialolithiasis in Slovakia with detailed information of the clinical intervention, histology, immunohistochemistry and elements content analysis.

## Case presentation

### Anamnesis and clinical findings

**Fig. 1:**
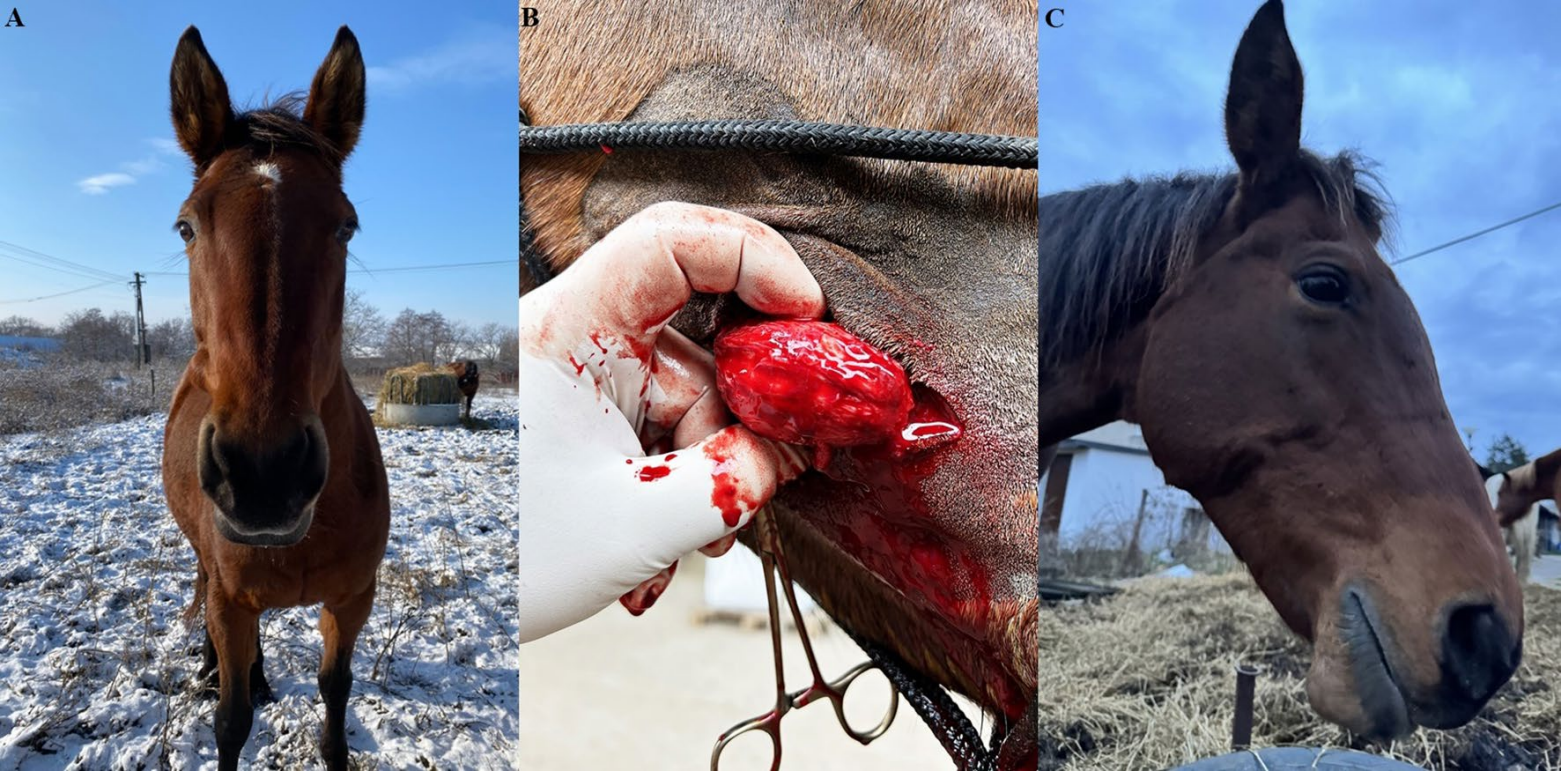
Frontal view on the mare with presence of hard mass on the right buccal region (**A**). Intraoperative photography of removing a hard mass from the cheek area by transcutaneous surgical approach (**B**). Lateral view on the buccal region without any reoccurrence of the sialolithiasis and presence of scars (**C**)

A 14-years-old Slovakian warmblood mare was presented with a hard, solid subcutaneous mass on the right side of the cheek. The size and form of the mass resembled a chicken egg (Fig. 1A). The mass was painless and did not cause any problems with food intake or using the horse for riding. The owner mentioned the mass first occurred 6 years ago, and continued growing for half a year since the first appearance. The dental examination under sedation showed normal and physiological dentition, without any dental issues. The presence of the mass did not cause the mare any problems during intake, chewing and swallowing of food. On the basis of a clinical and intraoral examination, sialolithiasis was suspected.

### Surgical procedure

A preliminary diagnosis of a single large sialolith in the rostral aspect of the right parotid salivary duct led to the decision to perform a surgical excision via transcutaneous approach under standing sedation. An intravenous catheter was aseptically placed in the right jugular vein. The horse was sedated intravenously with detomidine hydrochloride (Domidine 10 mg/ml, Eurovet Animal Health, B.V., Netherlands), 0.01 mg/kg BW, and butorphanol tartrate (Torbugesic Vet 10 mg/ml, Zoetis s.r.o., Czech Republic), 0.01 mg/kg BW. Anaesthesia was maintained with a constant rate infusion of a detomidine hydrochloride 0.02 mg/kg/hour and butorphanol tartrate 0.013 mg/kg/hours mixed in a 1 l bag of saline. Lidocaine hydrochloride (Lidocaine 2%, BIOPHARM, Výzkumný ústav biofarmacie a veterinárních léčiv a.s., Czech Republic) was percutaneously injected around the mass.

The right maxillary region was clipped and prepared aseptically for surgery. An incision was made over the mass with a #22-scalpel blade through the skin and subcutaneous tissues to expose an oval capsule (Fig. 1B). Enormous effort was made to avoid iatrogenic damage to the facial artery and vein located beside the proximal part of the parotid duct. The mass was gently removed, the parotid duct was catheterized with a canine urinary catheter and lavaged with saline to remove all sialoliths and tissue debris. The parotid duct was then ligated with polyglactin (Vicryl, Ethicon Inc., USA) USP 2/0 with a Cushing suture pattern. The subcutaneous tissue was apposed with a simple continuous pattern and the skin was apposed with a simple interrupted suture pattern. The oral aspect of the duct and oral mucosa were left to heal by secondary intention.

Post-operative medication included twice daily trimethoprim and sulfadiazine (Equibactin 45 g, Dechra Pharmaceuticals plc, UK) 30 mg/kg and twice daily phenylbutazone (Equipalazone, Dechra Pharmaceuticals plc, UK), 8.8 mg/kg, both administered for 5 days. No complications occurred during the postoperative period. The oral cavity was lavaged with 1% povidone iodine (Betadine, Egis Pharmaceuticals plc, Hungary) twice daily after meals for one week. The mare was re-examined 10 days after surgery to remove the skin sutures. The owner reported no clinical problems 6 months post-surgery, saliva production and swallowing remained physiological (Fig. 1C). No recurrence of the disease and no new deposits of sialoliths were observed.

### Gross morphological observation

**Fig. 2:**
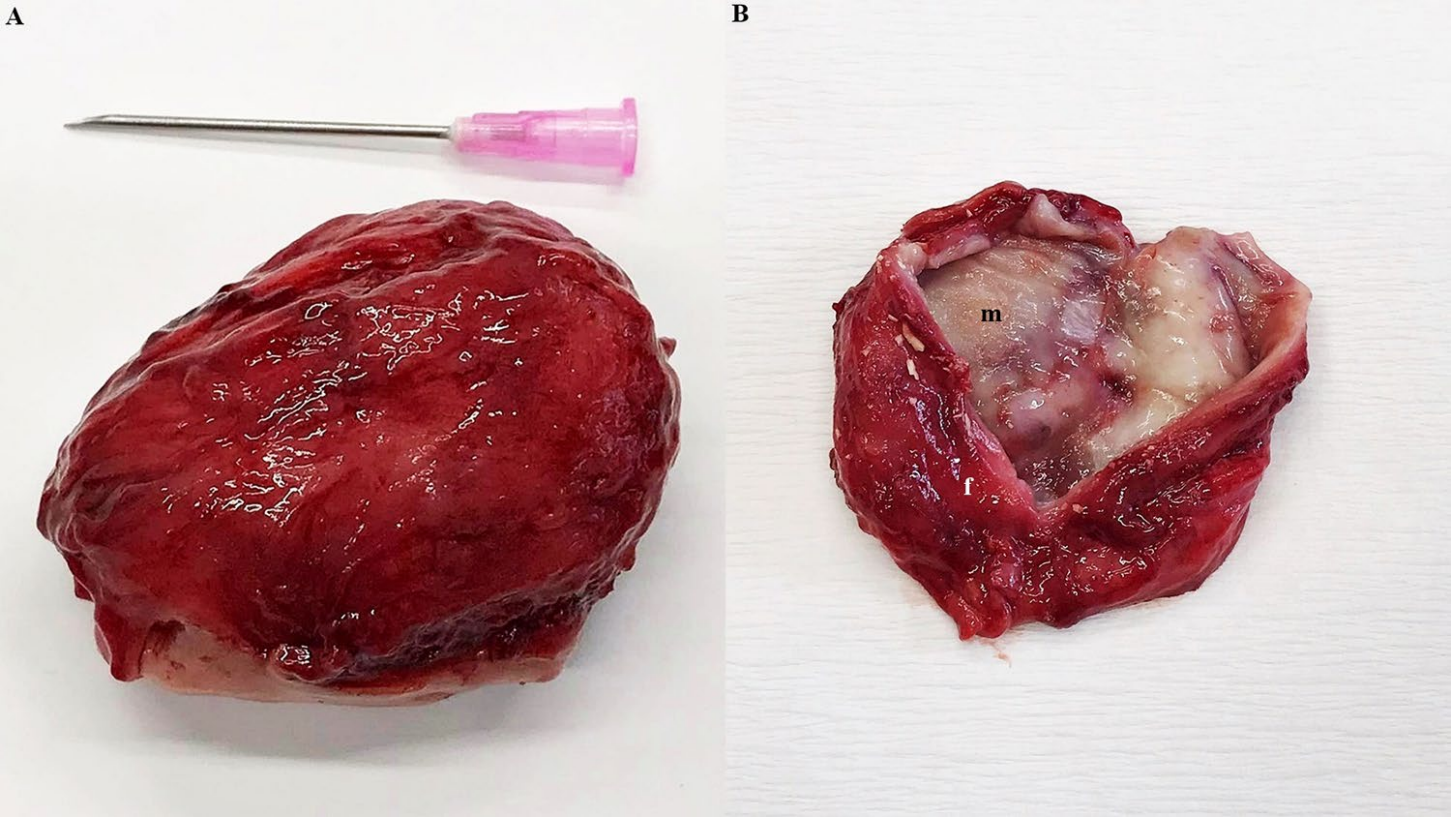
Extirpated mass—view on the external smooth well-vascular surface (**A**). The internal capsule organization presented the mucous membrane (m) and supportive (fibrous and muscular) (f) tissue (**B**), morphologically corresponds to ectatic parotid duct

**Fig. 3:**
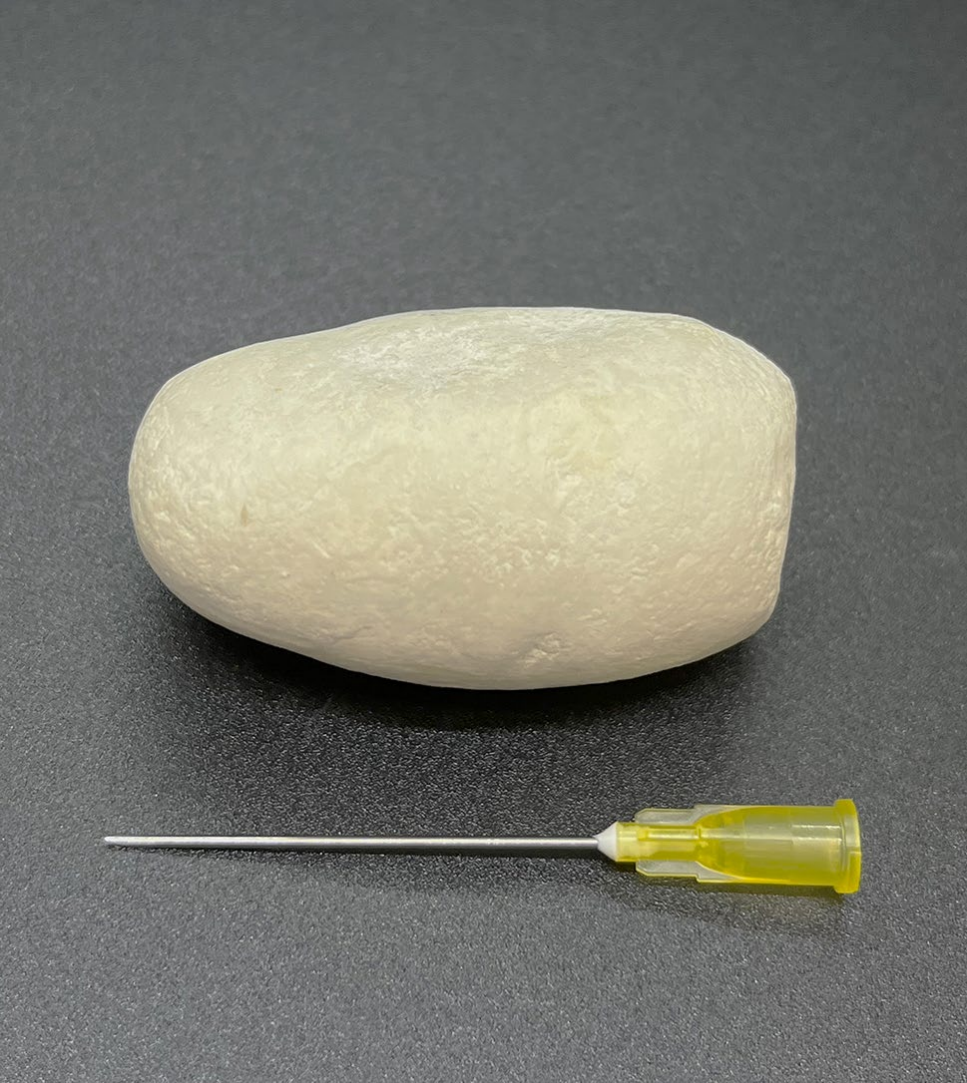
Sialolith removed from the ectatic parotid duct showing smooth surface without any erosions and sharp prominences, sized 49.88 × 30.94 × 25.10 mm, weighted 60.50 g

Incision into the capsule showed the presence of mucous membrane and fibrous tissue. The capsule was very well vascularized (Fig. 2). This capsule represented parotid duct ectasia in the terminal area. Inside the capsule was a well-organized white mineralized homogenous matter, with a smooth surface (Fig. 3). The sialolith´s dimensions were 49.88 × 30.94 × 25.10 mm, weighing 60.50 g.

## Histopathology

Tissues samples from the capsule were taken and fixed in 4% buffered formalin (Mikrochem Trade spol. s.r.o., Slovakia). Pieces of tissues were embedded in paraffin, sectioned into 5 μm thick sections and stained by routine hematoxylin-eosin and special Masson´s trichrome staining (both Diapath S.P.A., Italy). Mononuclear cells, presented by macrophages and lymphocytes were observed, which resulted in the use of immunohistochemistry to type the macrophages and plasma cells. An immunohistochemical method was carried out on paraffin-embedded tissue samples. Paraffin blocks were cut to 2 μm thick sections and revitalized by Ultra cell conditioning 1 medium in Ventana Benchmark Ultra automat (both Ventana Medical Systems, USA). Sections were incubated with monoclonal ready-to-use antibodies CD68 for macrophages (Ventana Medical Systems, USA) and CD138 for plasma cells (Dako-Agilent Technologies, Inc., USA). A positive colour reaction was visualized by UltraView DAB IHC Detection Kit (Ventana Medical Systems, USA). The samples were evaluated under an Olympus CX43 light microscope (Olympus, Japan), photos were taken by a PROMICAM 3-5CP + camera and processed via QuickPHOTO MICRO 3.2 software (both Promicra s.r.o., Czech Republic).

**Fig. 4:**
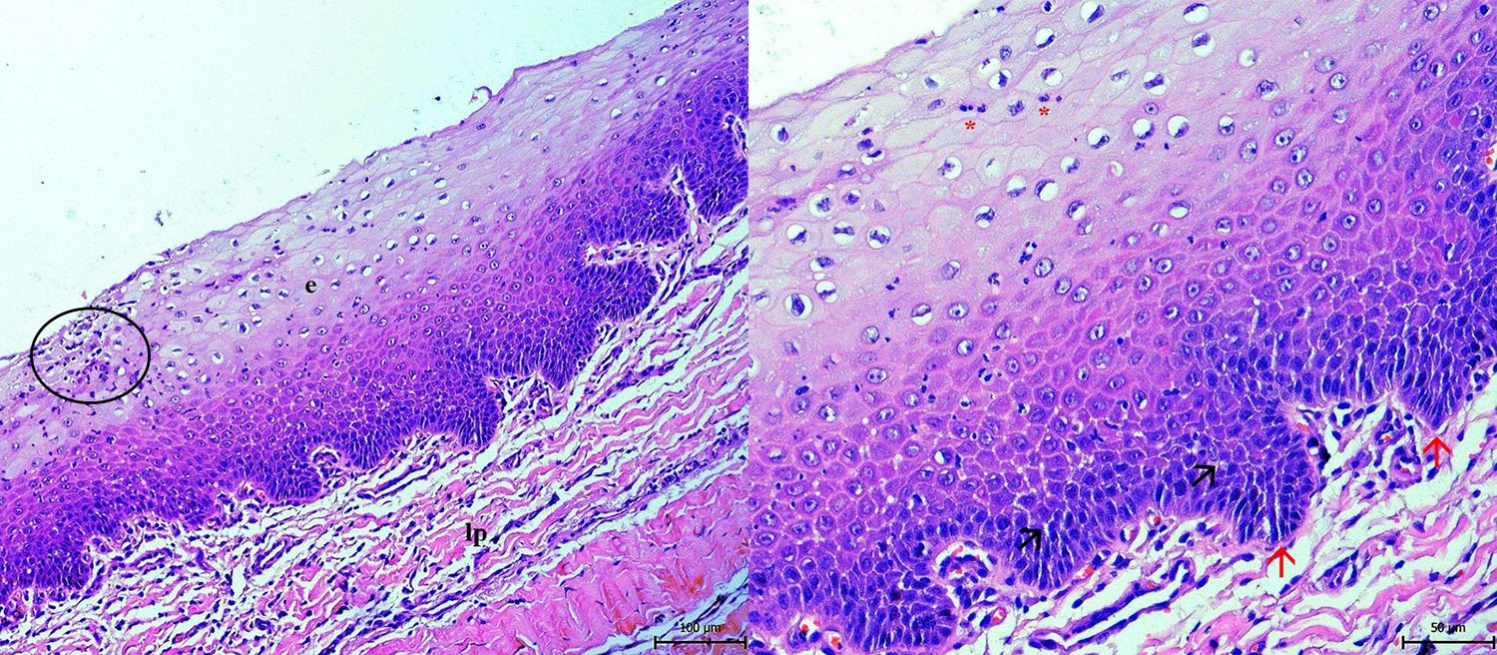
Mucous membrane of the ectatic parotid duct. Hyperplasia of epithelium (e) with spongiosis (arrows), acantosis (red arrows), focal epitheliolysis (circle) and neutrophilic exocytosis (asterisk). *Lamina propria mucosae* is present too (lp). Hematoxylin-eosin stain

**Fig. 5:**
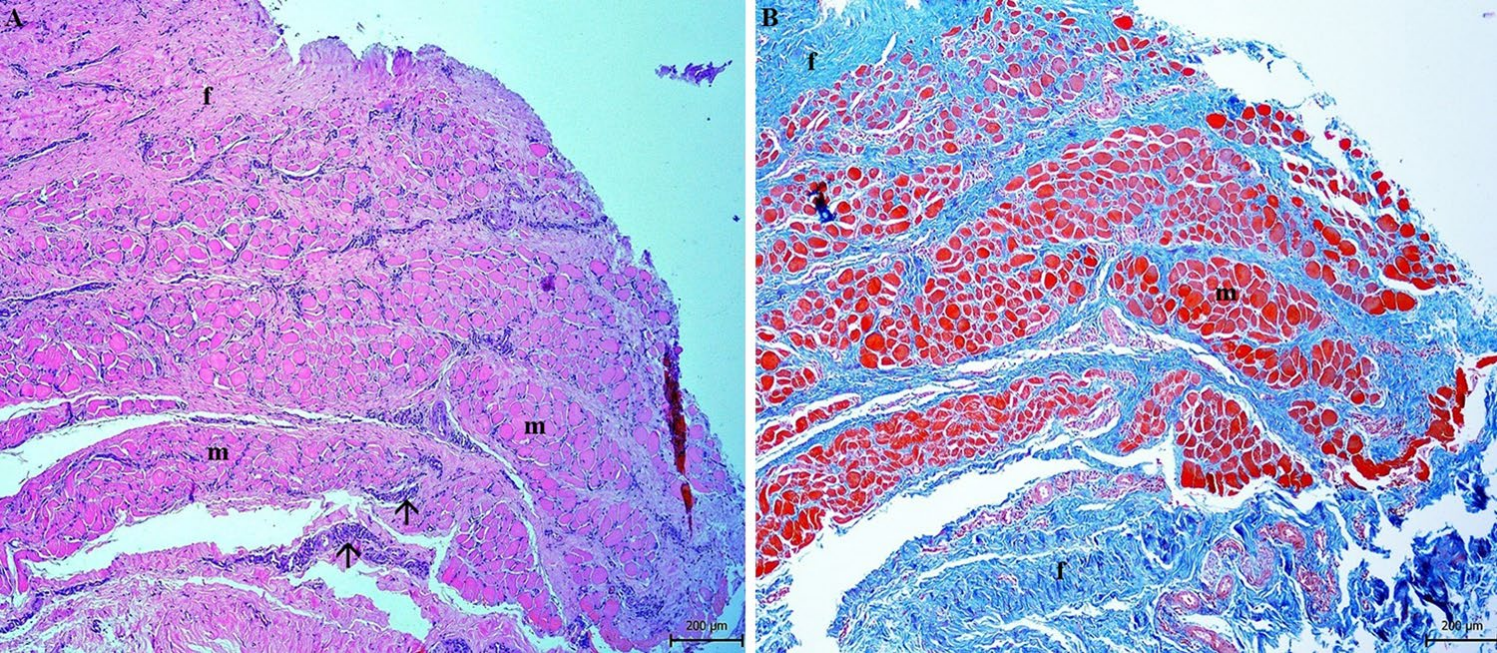
Presence of the muscular bundles (m) and fibrous tissue (f) in the wall of ectatic parotid duct (**A**, **B**). Proliferated collagenous fibers around muscles caused their atrophy (**B**). Cellular infiltrates (arrows) are localised between collagen fibers. **A** Hematoxylin-eosin stain, **B** Masson´s trichrome stain

**Fig. 6:**
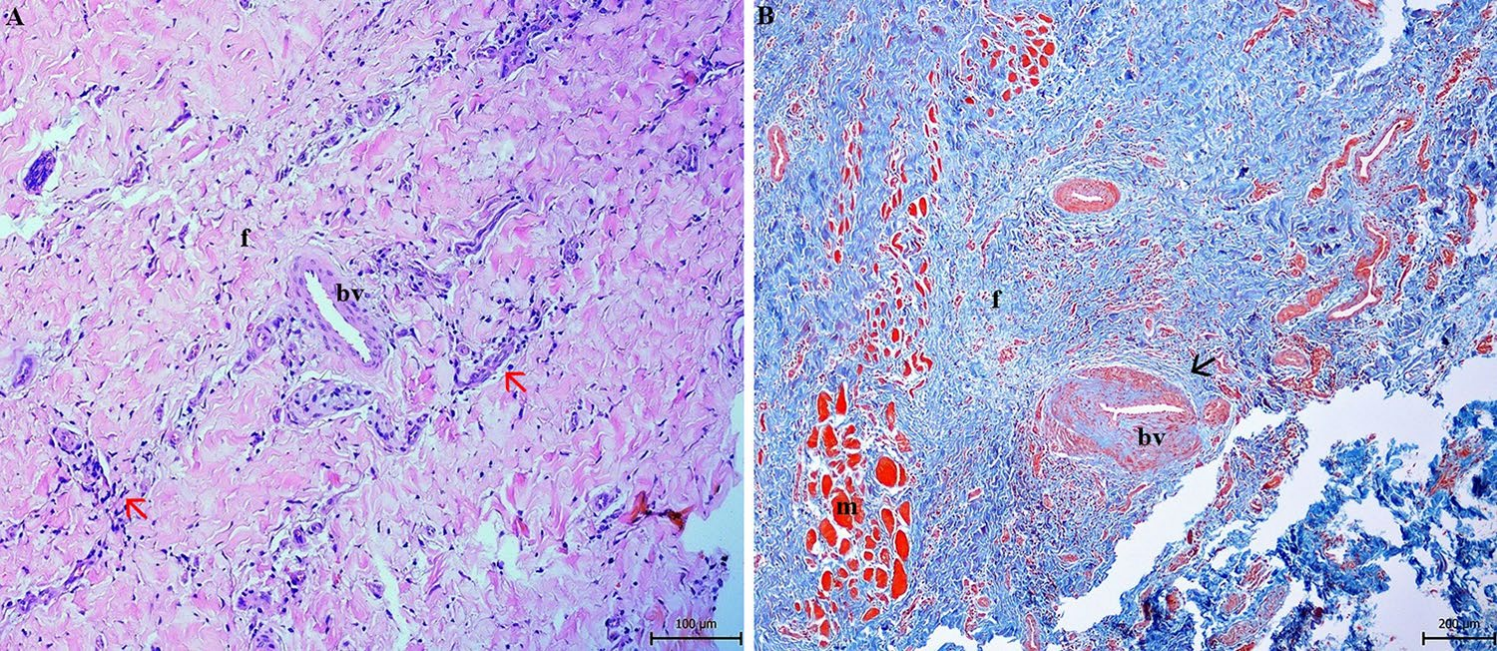
Presence of non-specific granulation tissue (f, m) with hyperplastic walls of blood vessels (bv) and perivascular fibrosis (arrow). Cellular infiltrates (red arrows) are localized around blood vessels. **A** Hematoxylin-eosin stain, **B** Masson´s trichrome stain

**Fig. 7:**
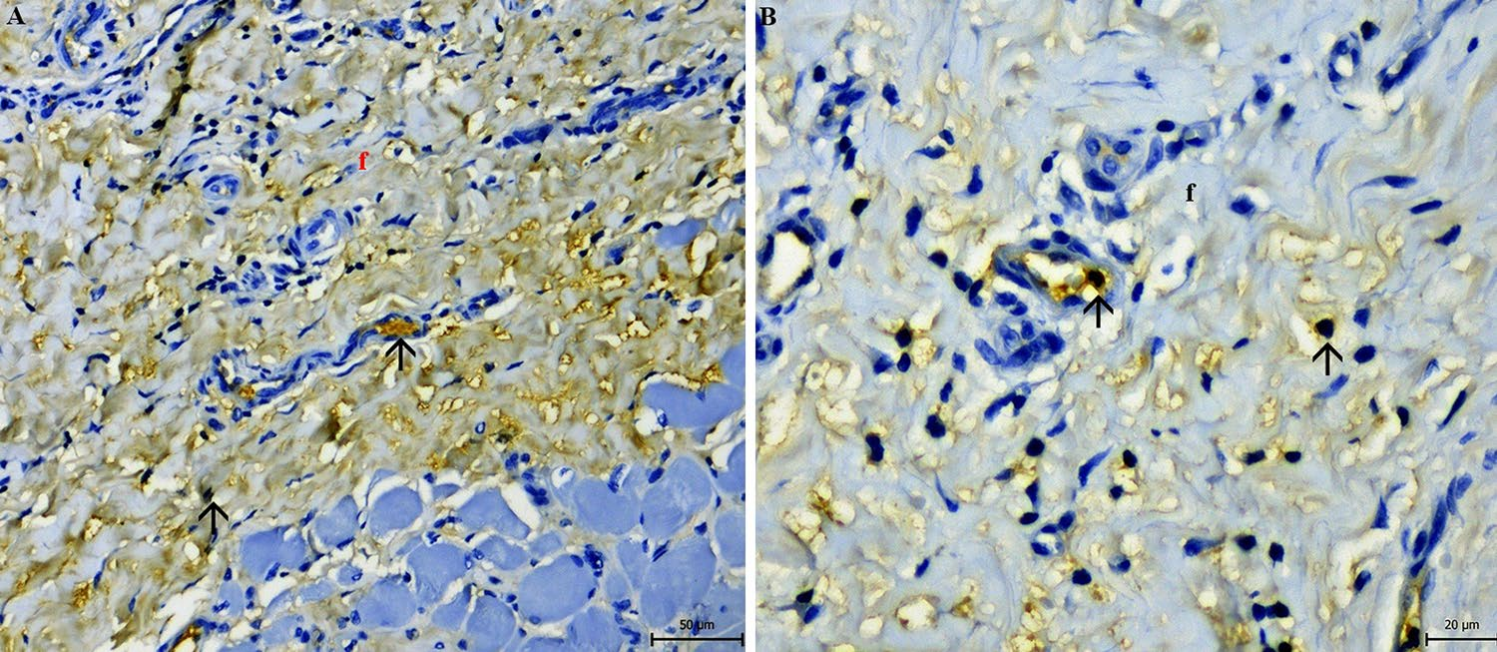
Immunohistochemical positivity of macrophages to CD68 antibody (**A**) and plasma cell to CD138 antibody (**B**) (arrows) in capsular fibrous tissue (f)

The histopathological examination confirmed the presence of hyperplastic epithelium with hyperkeratosis of the parotid duct mucous membrane. Examination of the epithelium showed acanthosis, spongiosis, intracellular oedema and epitheliolysis, with predominantly neutrophilic infiltration (Fig. 4). In the muscular and fibrous layer, granulation tissue with macrophages, plasma cells and fibroblasts were observed. Fibrous tissue was found to accumulate around and between muscle fibers (Figs. 5 and 6). The blood vessel walls were hyperplastic, with perivascular fibrosis. In the submucosal layer, mainly fibrous tissue was proliferated with focal mononuclear infiltrates disseminated mainly perivascularly (Figs. 5 and 6). Immunohistochemistry showed the mononuclear infiltrates consisted of macrophages, demonstrated by a strong positive response to CD68 antibody. Sporadic occurrence of CD138 positive plasma cells was demonstrated by a weak positive reaction (Fig. 7).

### Element content analysis

Dry samples of the sialolith were processed with a digestive method, in a microwave oven MLS-1200 Mega (Milestone s.r.l., Italy), using 5 ml HNO_3_ and 1 ml HCl per 1 g of sample (Mikrochem Trade spol. s.r.o., Slovakia). The process of digestion was completed in 6 steps as follows: 1st 250 W 2 min, 2nd 0 W 2 min, 3rd 250 W 5 min, 4th 400 W 5 min, 5th 500 W 5 min, 6th 600 W 2 min. The digested samples of sialolith were analysed for the presence of calcium, magnesium, sodium, potassium, copper and zinc by flame atomic absorption spectroscopy with a Unicam 939 Solar (Unicam Ltd, UK). The methodology presented in the List of Official Methods and Laboratory Diagnostics of Food and Feed (Bulletin of the Ministry of Agriculture SR, 2004) was used for determination of the composition. The content of phosphorus was analysed by the colorimetric method. Absorbance was measured with a spectrophotometer Visible V 5000 (Shanghai Metash Instruments Co., Ltd., China) at a wavelength of λ = 666 nm. Chlorine concentration was determined by the commercial test CL 250 (Erba Lachema s.r.o., Czech Republic). Absorbance was measured with a spectrophotometer Specord 210 Plus (Analytik Jena AG, Germany) at a wavelength of λ = 492 nm.

The atomic absorption spectroscopy of the sialolith confirmed a high content of calcium and trace amounts of the other analysed elements. A detailed description of the elemental composition of the sialolith is described in Table 1.

**Table 1:** Macro and micro elements content in the sialolith

Element	Content	Unit
Calcium	252.00	g kg^−1^
Phosphorus	4.60	g kg^−1^
Magnesium	1.60	g kg^−1^
Sodium	0.80	g kg^−1^
Potassium	0.01	g kg^−1^
Copper	29.00	mg kg^−1^
Zinc	47.30	mg kg^−1^
Chlorine	111.19	mmol l^−1^

### Mineral composition analysis

The mineral composition of the sialolith was analysed with X-ray powder diffraction (XRD). XRD analysis was conducted using a Panalytical X´Pert PRO MPD diffractometer (Malvern Panalytical Ltd., UK) by reflection geometry equipped with a cobalt tube (λKα = 0.17903 nm), iron (Fe) filter and 1-D RTMS (X’Celerator) detector. The procedure included a step size of 0.033° 2Θ, time per step of 160 s, an angular range of 4–100° 2Θ, and a total scan duration of 3701 s. The acquired data was evaluated using the Panalytical HighScore 5.1 plus software (Malvern Panalytical Ltd, UK).

**Fig. 8:**
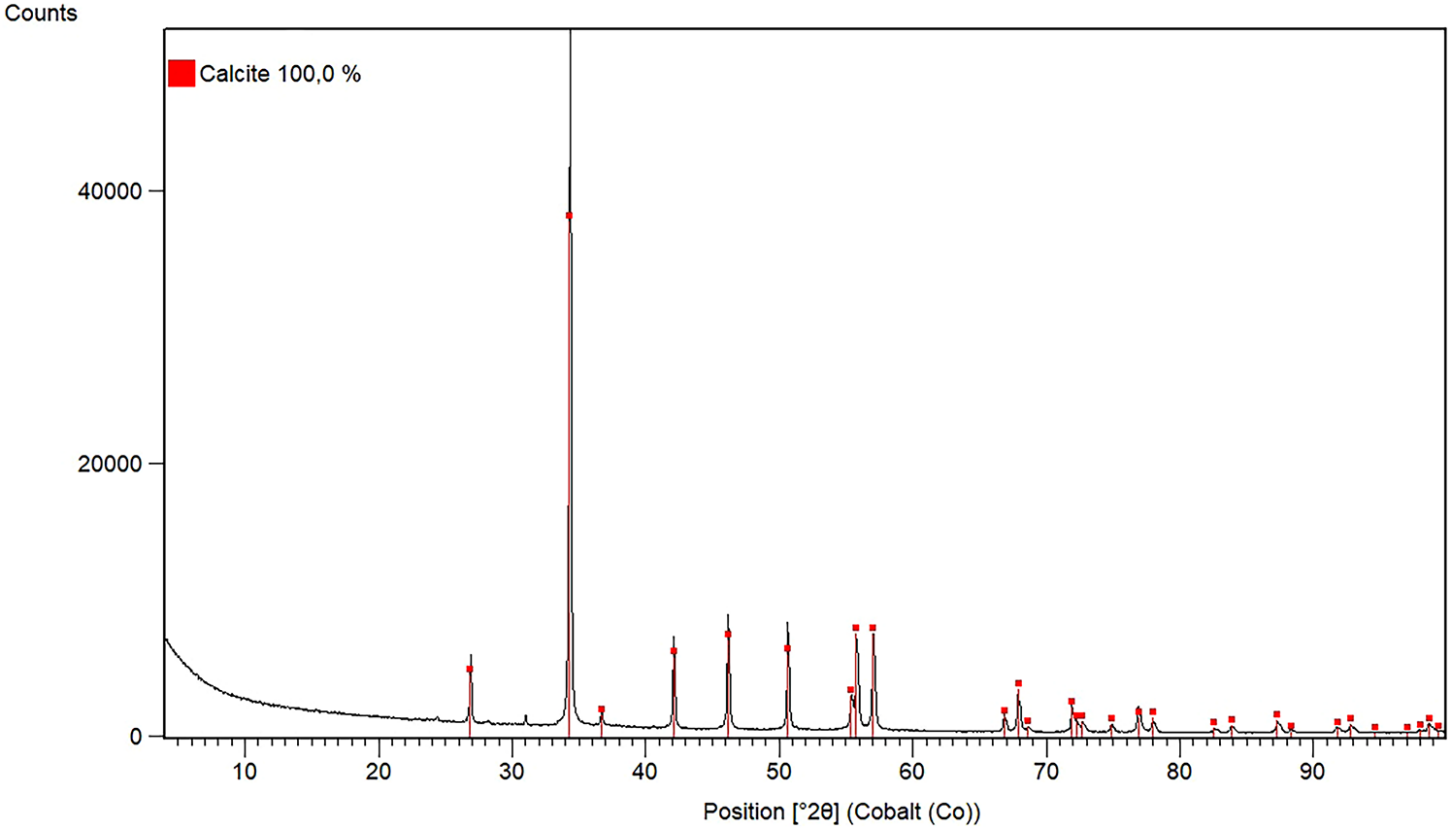
Red lines mark positions of calcite reflections from database (ICSD, Record No. 28827)

The XRD analysis showed that the only crystalline phase present in the sample was composed of calcite (calcium carbonate, CaCO_3_) (Fig. 8).

## Discussion

Sialolithiasis is an uncommon condition that has been well described in dogs, equids (horses and donkeys), ruminants and humans (Trumpatori et al. 2007; Rodrigues et al. 2013; Misk et al. 2014; Han et al. 2020; Kraaij et al. 2023). Possible explanations for the formation of sialoliths in humans include hyposalivation, dehydration, changes in biochemical saliva composition, decreased solubility of crystalloids, and/or anatomical variation of the canalicular systems and ducts (Nagra et al. 2010; Avishai et al. 2021; Kraaij et al. 2023). In animals, there is no satisfactory explanation for the formation and aetiology of sialoliths. We could assume that the same causes for sialolith formation in humans play the same role in animals. However, most authors describing sialolith formation in horses, describe the formation of sialolith as a response to the penetration of an organic nidus into the interior of the parotid duct, causing inflammation and subsequent accumulation of minerals (Kay 2006; Al-Sobayil and Ibrahim 2008; Oreff et al. 2016; Poore et al. 2019). Occurrence of sialolithiasis in horses is reported most often in the parotid duct, whereas in humans, occurrence is most often reported in the submandibular gland or duct (72–95%) (Kraaij et al. 2023).

A physiologically normal parotid duct in horses has diameter of 3.0–3.9 mm according to measurements taken during sialography (Dehghani et al. 2005). This case reported parotid duct ectasia due to obstruction by a 4.9 × 3.0 × 2.5 cm sialolith. Other studies have reported sialoliths between 6 and 13 cm long (Kay 2006; Al-Sobayil and Ibrahim 2008; Rodrigues et al. 2013; Oreff et al. 2016; Aoyama et al. 2018; Poore et al. 2019; Diakakis and Karadina 2021).

There are two possible approaches to remove a sialolith—transcutaneous and transoral approaches (MacLean 2006; Carlson et al. 2015). Both methods represent a fast solution to the problem—neither method requires general anaesthesia or recumbence of the horse, and can be performed in the field (Rodrigues et al. 2013; Oreff et al. 2016). The transoral approach is frequently used to remove sialoliths, when the sialolith is “visible” through oral cavity. There are two ways of sialolith removal through the oral cavity. Incision of the mucosa of the parotid duct, or “opening” of the parotid papilla (Kay 2006; Oreff et al. 2016). Incision into the parotid duct mucosa is a faster method as there is no needed to localize the parotid papilla (Oreff et al. 2016). Frequently observed complications of the transoral approach include focal cellulitis, infection, fistulas and granulation tissue formation (Carlson et al. 2015; Oreff et al. 2016). The transcutaneous approach is chosen in the case of large sialoliths (over 6 cm). According to some authors, it is a simpler technique, but the possibility of complications is much higher than the transoral approach (Kay 2006; Al-Sobayil and Ibrahim 2008; Rodrigues et al. 2013; Aoyama et al. 2018; Poore et al. 2019). Possible complications connected with transcutaneous approach include the formation of oro-cutaneous fistulas, iatrogenic damage to the buccal branches of the facial nerve, damage to the facial artery and vein, the infection and oedema around the incision (Kay 2006; MacLean 2006; Carlson et al. 2015). In this case, the transcutaneous approach was chosen due to easier and faster access on a standing horse. There were no complications, nor damage to clinically important vessels and nerves in the face/cheek area. Currently, the transoral approach is preferred and recommended by surgeons, as the complications are minimized during surgery and healing.

Histopathological examination of the parotid duct revealed ectasia with hyperplastic epithelial transformation. Rich vascularized granulation tissue which included plasma cells and macrophages in ductal connective tissue were found, likely resulting from long term presence and chronic irritation by the sialolith. Trumpatori et al. (2007), who performed histological analysis of an ectatic parotid duct in a dog, reported similar results. According to our best knowledge, detailed information about histology of parotid duct ectasia in horses is lacking.

Macro- and microelement composition of sialoliths in horses has been analysed by a single study. Aoyama et al. (2018) analysed sialoliths and expressed their mineral content in percentage. Similarly to this study, it was reported that sialoliths were predominantly composed of calcium and phosphorus. Aoyama et al. (2018) however, did not determine the type of mineral with X-ray powder diffraction. In humans, elemental analysis has been performed in two studies. Im et al. (2017) performed detailed chemical analysis of submandibular gland sialoliths by employing energy dispersive X-ray spectroscopy (EDS). Different areas of the sialoliths showed diverse chemical content, with varying ratios of calcium to phosphorus. The major elements with the highest content were carbon, nitrogen, oxygen, the smallest amount represented was magnesium and sulphur. Another study, Sadaksharam and Kuduva Ramesh (2020) analysed three sialoliths (2 from the parotid and 1 from the submandibular duct) by EDS, revealing differences in the organic and inorganic composition of parotid and submandibular duct stones. Organic substances comprised 15% of the submandibular duct sialolith, compared to 40% of the parotid duct sialoliths. Inorganic substances in the submandibular duct sialolith represented 85% of the overall composition, compared to 60% of the parotid duct sialolith. The content of calcium and phosphorus and their mutual ratio also differed. The parotid duct sialolith contained 10% calcium and 5.9% phosphorus, the submandibular duct sialolith had higher percentage of calcium with 28% as well as 16.8% of phosphorus.

There are mineral composition differences in animals and human sialoliths. Hydroxyapatite, amorphous carbonated calcium phosphate, carbonate apatite, octocalcium phosphate pentahydrate, tricalcium phosphate and whitlockite are frequent components of sialoliths in human (Sabot et al. 2012; Kraaij et al. 2020). In animals, mainly horses and dogs, in which few studies reported detailed mineral content analyses, the predominant minerals in sialoliths included calcium carbonate (Schumacher and Schumacher 1995; Kay 2006; Trumpatori et al. 2007; Oreff et al. 2016; Diakakis and Karadima 2021), apatite (Oreff et al. 2016), mixture of the calcium carbonate with calcium phosphate (Tivers and Moore 2007; Poore et al. 2019), and mixture of calcium carbonate with calcium oxalate (Al-Sobayil and Ibrahim 2008). Among the less represented minerals were hydroxyapatite, magnesium carbonate, magnesium ammonium phosphate. These minerals occurred in various mixtures (Kay 2006; Trumpatori et al. 2007).

## Conclusion

Sialolithiasis is a very uncommon disease in horses, and this is the first reported case of sialolithiasis in Slovakia. Although the transoral approach of sialoliths extirpation from the parotid duct is currently recommended, the transcutaneous approach on a standing horse was preferred for easier execution. Pathological examination confirmed the suspected diagnosis of sialolithiasis in an ectatic parotid duct changed by granulation tissue. The horse’s reconvalescence was good and no other problems or disease recurrence have been reported after almost 2 years post-surgery. The sialolith was composed of calcite (calcium carbonate) detected by XRD. Based on the disease uniqueness, similar analytical studies using multiple interdisciplinary methodologies are necessary for a correct understanding of the disease aetiology and setting aimed treatment or prevention.

## Data Availability

No datasets were generated or analysed during the current study.

## References

[CR1] Al-Sobayil FA, Ibrahim IM (2008) Parotid duct sialolithiasis in horses. J Equine Vet Sci 28:437–439. 10.1016/j.jevs.2008.06.002

[CR2] Aoyama IHA, Campebell RDC, Zambrano RS et al (2018) Sialolitíase em equino: relato de caso. Arq Bras Med Vet Zootec 70:353–358. 10.1590/1678-4162-9884

[CR3] Avishai G, Ben-Zvi Y, Chaushu G et al (2021) The unique characteristics of sialolithiasis following drug-induced hyposalivation. Clin Oral Investig 25:4369–4376. 10.1007/s00784-020-03750-2 33389134 10.1007/s00784-020-03750-2

[CR4] Carlson N, Eastman T, Winfield L (2015) Sialolithiasis in horses: a retrospective study of 25 cases (2002–2013). Can Vet J 56:1239–1244 26663918 PMC4668825

[CR5] Dehghani SN, Tadjalli M, Seifali A (2005) Sialography in horse: technique and normal appearance. Vet Arh 75:531–540

[CR6] Diakakis N, Karadima V (2021) Equine sialolithiasis of the distal parotid duct. Retrospective study on 4 cases. Arch Vet Sci Med 4:13–23. 10.26502/avsm.021

[CR7] Han HJ, Mann FA, Yoon HY (2020) Parotid duct ectasia in a dog. J Am Anim Hosp Assoc 56:48–52. 10.5326/jaaha-ms-6753 31715117 10.5326/JAAHA-MS-6753

[CR8] Im YG, Kook MS, Kim BG et al (2017) Characterization of a submandibular gland sialolith: micromorphology, crystalline structure, and chemical compositions. Oral Surg Oral Med Oral Radiol 124:13–20. 10.1016/j.oooo.2017.03.011 10.1016/j.oooo.2017.03.011 28483473

[CR9] Kay G (2006) Sialolithiasis in equids, a report on 21 cases. Equine Vet Educ 18:333–336. 10.1111/j.2042-3292.2006.tb00472.x

[CR10] Kraaij S, de Visscher JG, Apperloo RC et al (2023) Lactoferrin and the development of salivary stones: a pilot study. Biometals 36:657–665. 10.1007/s10534-022-00465-7 36396778 10.1007/s10534-022-00465-7 PMC10181970

[CR11] MacLean YT (2006) Chronic sialolithiasis in a Trakehner mare. Can Vet J 47:480–482 16734376 PMC1444901

[CR12] Misk NA, Misk TN, Semieka MA et al (2014) Affections of the salivary ducts in buffaloes. Open Vet J 4:65–68. 10.5455/ovj.2014.v4.i1.p65 26623341 PMC4629587

[CR13] Nagra I, Jones C, Dyer J (2010) Endoluminal intervention in the salivary duct: clinical outcomes at a district general hospital. CardioVasc Interv Radiol 33:307–314. 10.1007/s00270-009-9731-3 10.1007/s00270-009-9731-3 19937028

[CR14] Oreff GL, Shiraki R, Kelmer G (2016) Removal of sialoliths using the intraoral approach in 15 horses. Can Vet J 57:647–650 27247466 PMC4866672

[CR15] Orsini JA, Grenager NS, de Lahunta A (2022) Comparative veterinary anatomy: a clinical approach, 1st edn. Academic, Cambridge

[CR16] Poore L, Smit Y, Williams J et al (2019) Standing transcutaneous surgical excision of a sialolith in an 11-year-old Thoroughbred mare. Equine Vet Educ 31:343–347. 10.1111/eve.12864

[CR17] Rodrigues JB, Mora S, Bastos E et al (2013) Percutaneous approach for sialolith removal in a donkey. J Vet Dent 30:30–33. 10.1177/089875641303000104 23757823 10.1177/089875641303000104

[CR18] Sabot JF, Gustin MP, Delahougue K et al (2012) Analytical investigation of salivary calculi, by mid-infrared spectroscopy. Analyst 137:2095–2100. 10.1039/c2an15924d 22416268 10.1039/c2an15924d

[CR19] Sadaksharam J, Kuduva Ramesh SS (2020) Spectroscopic and microscopic characterisation of parotid and submandibular ductal sialoliths: a comparative preliminary study. Proc Natl Acad Sci India Sect B Biol Sci 90:1165–1171. 10.1007/s40011-020-01189-9

[CR20] Schumacher J, Schumacher J (1995) Diseases of the salivary glands and ducts of the horse. Equine Vet Educ 7:313–319. 10.1111/j.2042-3292.1995.tb01257.x

[CR21] Singh B (2017) Dyce, Sack and Wensing’s textbook of veterinary anatomy. Saunders, Philadelphia

[CR22] Tivers MS, Moore AH (2007) Surgical treatment of a parotid duct sialolith in a bulldog. Vet Rec 161:271–272. 10.1136/vr.161.8.271 17720965 10.1136/vr.161.8.271

[CR23] Trumpatori BJ, Geissler K, Mathews KG (2007) Parotid duct sialolithiasis in a dog. J Am Anim Hosp Assoc 43:45–51. 10.5326/0430045 17209085 10.5326/0430045

